# Pharmacological Interventions for the MATRICS Cognitive Domains in Schizophrenia: What’s the Evidence?

**DOI:** 10.3389/fpsyt.2013.00157

**Published:** 2013-12-04

**Authors:** Wilhelmina A. M. Vingerhoets, Oswald J. N. Bloemen, Geor Bakker, Therese A. M. J. van Amelsvoort

**Affiliations:** ^1^Department of Psychiatry and Psychology, Maastricht University, Maastricht, Netherlands; ^2^Department of Nuclear Medicine, Academic Medical Centre, University of Amsterdam, Amsterdam, Netherlands

**Keywords:** pharmacology, schizophrenia, cognition, MATRICS, neurotransmitters

## Abstract

Schizophrenia is a disabling, chronic psychiatric disorder with a prevalence rate of 0.5–1% in the general population. Symptoms include positive (e.g., delusions, hallucinations), negative (e.g., blunted affect, social withdrawal), as well as cognitive symptoms (e.g., memory and attention problems). Although 75–85% of patients with schizophrenia report cognitive impairments, the underlying neuropharmacological mechanisms are not well understood and currently no effective treatment is available for these impairments. This has led to the Measurement and Treatment Research to Improve Cognition in Schizophrenia (MATRICS) initiative, which established seven cognitive domains that are fundamentally impaired in schizophrenia. These domains include verbal learning and memory, visual learning and memory, working memory, attention and vigilance, processing speed, reasoning and problem solving, and social cognition. Recently, a growing number of studies have been conducted trying to identify the underlying neuropharmacological mechanisms of cognitive impairments in schizophrenia patients. Specific cognitive impairments seem to arise from different underlying neuropharmacological mechanisms. However, most review articles describe cognition in general and an overview of the mechanisms involved in these seven separate cognitive domains is currently lacking. Therefore, we reviewed the underlying neuropharmacological mechanisms focusing on the domains as established by the MATRICS initiative which are considered most crucial in schizophrenia.

## Introduction

Schizophrenia is a disabling, chronic psychiatric disorder with a prevalence rate of 0.5–1% in the general population. Symptoms include positive and negative symptoms and disorganization. Approximately 75–85% of the schizophrenia patients report cognitive impairments as well (1). Although this aspect of schizophrenia was already described by Kraepelin nearly a century ago, cognitive impairments have long been under-identified as a symptom of schizophrenia and relatively little research has been conducted on this topic. Previous research showed that cognitive functioning is strongly associated with functional outcome in schizophrenia [e.g., skill acquisition in psychosocial rehabilitation treatment, demonstration of ability to solve simulated interpersonal problems, and community functioning (2, 3)]. Cognitive impairments often precede the onset of other symptoms and persist after other psychotic symptoms have been effectively treated (4). Furthermore, severity of cognitive impairments is predictive of poorer medication compliance (5), treatment adherence (6), and increased tendency for relapse in first-episode patients (7). At present, no effective treatment is available as existing (antipsychotic) medication mainly targets positive symptoms and does not improve cognition. Although it is sometimes assumed by clinicians (possibly due to marketing) that atypical antipsychotics are superior to typical antipsychotics, results from recent studies contradict this theory. Presently available guidelines for treatment of schizophrenia, such as the NICE (8), PORT (9), and WFSBP (10) guidelines, indicate that there is no superiority of atypical antipsychotics on positive symptoms with the exception of clozapine, and instead state that use of antipsychotics should be evaluated based on their side effects. Since cognitive dysfunction is associated with functional outcome, development of an effective intervention strategy for these symptoms and corresponding guidelines is essential, as such guidelines are currently still lacking.

Lack of effective treatment strategies has over recent years led to an increase in studies investigating underlying neurobiological mechanisms of cognitive impairments and potential new pharmacological targets to enhance cognition in schizophrenia. Research has mainly focused on the role of neurotransmitters such as dopamine, serotonin, γ-aminobutyric acid (GABA), glutamate, and acetylcholine. Previous research indicated that specific cognitive impairments seem to arise from different underlying neurobiological mechanisms (11). For example, the prefrontal cortex (PFC) has been implicated in the executive functioning aspect of cognition (12) whereas the hippocampus has been linked to, e.g., episodic memory (13). This suggests that specific pharmacological agents could enhance domains of cognition differentially. Nonetheless, still little is known about the underlying neurobiology of cognition. Knowledge about these neurobiological mechanisms is highly necessary for development of new pharmacological intervention strategies.

To improve cognition research in schizophrenia the Measurement and Treatment to Improve Cognition in Schizophrenia (MATRICS) was developed. The MATRICS identified seven cognitive domains that are fundamentally impaired in schizophrenia: verbal learning and memory, visual learning and memory, working memory, attention and vigilance, processing speed, reasoning and problem solving, and social cognition (14). It was decided that cognition research in schizophrenia should mainly focus on these domains in order to identify the neurobiological mechanisms, ultimately to facilitate development of new pharmacological treatment strategies.

Although specific domains of cognition have been identified, most studies tend to describe cognition in general terms using a composite score. Currently, a review differentiating between separate MATRICS domains is lacking. Therefore, the aim of this review is to provide an outline of the underlying neuropharmacological mechanisms of each individual cognitive domain. We will focus on pharmacological intervention studies which used validated instruments to measure the effect on the MATRICS cognitive domains.

## Methods

### Search strategy

A literature search was conducted in medical database PubMed. The following keywords were used: “pharmacology,” “schizophrenia,” and “Cognition.” Subsequently, a separate search was conducted for each individual domain combining the keywords “pharmacology” and “schizophrenia” with the following keywords: “verbal learning,” “verbal memory,” “visual learning,” “visual memory,” “working memory,” “attention,” “vigilance,” “processing speed,” “reasoning,” “problem solving,” and “social cognition.”

### Inclusion and exclusion criteria

Papers that met the following inclusion criteria were included: (1) original research papers, both single challenge and clinical trials (full text available); (2) published in English; (3) use of a pharmacological intervention (pharmacological interventions used and their main mechanism of action are displayed in Table 1); (4) use of validated cognitive tests to measure one or more of the MATRICS domains; (5) subjects were patients with schizophrenia; and (6) were published between January 2000 and May 2013. Papers were excluded if: (1) only healthy subjects were included; (2) also schizoaffective disorders and other psychotic disorders were included; (3) cognitive domains were measured with non-validated tests; (4) cognitive domains other than the MATRICS domains were measured; and (5) only a composite score of cognition was reported.

**Table 1:** **Pharmacological agents used and their main mechanism of action**.

General pharmacological domain	Pharmacological agent	Agents main mechanism of action
Antipsychotics	Haloperidol	D_2_ antagonist
	Chlorprothixene	D_2_ antagonist
	Perazine	D_2_ antagonist
	Flupenthixol	D_2_ antagonist
	Amisulpride	D_2_/D_3_ antagonist
	Risperidone	D_2_ and 5-HT_2a_ antagonist
	Paliperidone	D_2_ and 5-HT_2a_ antagonist (active metabolite of risperidone)
	Olanzapine	D_2_ and 5-HT_2a_ antagonist
	Loxapine	D_2_ and 5-HT_2a_ antagonist
	Sertindole	D_2_ and 5-HT_2a_ antagonist
	Ziprasidone	D_2_ and 5-HT_2a_ antagonist
	Quetiapine	D_2_ and 5-HT_2a_ antagonist partial 5-HT_1a_ agonist
	Aripiprazole	5-HT_2a_ antagonist and partial D_2_ agonist and partial 5-HT_1a_ agonist
	Perospirone	D_2_ and 5-HT_2a_ antagonist and 5-HT_1a_ agonist
	Clozapine	D_2_, D_4_, and 5-HT_2a_ antagonist partial 5-HT_1a_ agonist (metabolite shows weak partial agonistic effects on D_2_ and D_3_)
	BL-1020	D_2_ and 5-HT_2a_ antagonist GABA_A_ agonist
Dopaminergic	Dihydrexidine	D_1_ agonist
Serotonergic	Ondansetron	5-HT_3a_ antagonist
	Tandospirone	5-HT_1a_ agonist
	Tropisetron	5-HT_3a_ antagonist
	Fluvoxamine	Reuptake inhibition and sigma-1 receptor agonist
	Citalopram	Reuptake inhibition
Cholinergic	AZD3480	nicotinic α_4_β_2_ agonist
	DMXB-A	Partial nicotinic α_7_ agonist
	Donepezil	Acetylcholinesterase inhibition
	Galantamine	Acetylcholinesterase inhibition
	Rivastigmine	Acetylcholinesterase inhibition
Glutamatergic	Minocycline	(Mainly used as antibiotic) blocks nitric oxide induced neurotoxicity
	d-Cycloserine	NMDA partial agonist
	Lamotrigine	Glutamate release regulation
	Topiramate	AMPA/kainite antagonist
GABAergic	MK-0777	GABA α_2_/α_3_ agonist
	Lorazepam	GABA_A_ agonist
	Flumazenil	GABA_A_ antagonist
Noradrenergic	Atomoxetine	Reuptake inhibition
Stimulants	d-Amphetamine	Dopamine/norepinephrine releaser thus indirect dopamine agonist
	Modafinil	Unknown
	Armodafinil	Unknown
Other domains	Mifepristone	Glucocorticoid receptor antagonist
	Sildenafil	PDE5 inhibition

## Results

In total, the search strategy yielded 938 articles of which 293 articles were found using the keywords “pharmacology,” “cognition,” and “schizophrenia.” The separate searches yielded 158 articles for verbal learning and memory, 85 for visual learning and memory, 100 for working memory, 234 for attention and vigilance, 22 for processing speed, 17 for reasoning and problem solving, and 29 for social cognition. After final screening 44 articles were included for verbal learning and memory, 26 for visual learning and memory, 43 for working memory, 22 for attention and vigilance, 31 for processing speed, 30 for reasoning and problem solving, and 7 for social cognition. Study characteristics and specifics are shown in Table 2 (studies investigating antipsychotics) and Table 3 (studies investigating non-antipsychotic intervention strategies).

**Table 2:** **Summary of included studies using antipsychotics**.

Study	Agent(s)	Sample size (in analyses)	Design	MATRICS domains measured	Main results
Wagner et al. (16)	Olanzapine and amisulpride	*N* = 36	8-week randomized clinical trial	Ver. l and m; wm; attention; ps	No difference between treatments conditions in ver. l and m, wm, attention, and ps. Both the olanzapine and the amisulpride group improved on ver. l and m and wm. Attention and ps did not improve in both treatment conditions
Tyson et al. (17)	Risperidone, olanzapine, clozapine, quetiapine, and amisulpride	*N* = 44	18-month clinical trial	Vis. l and m; wm; r and ps	Vis. l and m, wm, and r and ps improved in the low affinity group (quetiapine and amisulpride) whereas vis. l and m and r and ps declined in the high-affinity group (risperidone, olanzapine, and clozapine)
Purdon et al. (20)	Risperidone, olanzapine, and haloperidol	*N* = 55	54-week randomized, double-blind trial	Ver. l and m; vis. l and m; wm; ps; r and ps	After Bonferroni correction, none of the cognitive domains improved in any of the treatment conditions
Malhotra et al. (21)	Ziprasidone	*N* = 10	12-week open-label clinical trial	Ver. l and m; ps	Significant improvement in ver. l and m. Ps did not improve significantly
Rémillard et al. (22)	Risperidone and haloperidol	*N* = 28	12-month randomized	Ver. l and m	Both types of antipsychotics did not improve ver. l and m
Harvey et al. (23)	Quetiapine and risperidone	*N* = 289	8-week, randomized, double-blind clinical trial	Ver. l and m; attention; ps	Both treatment conditions improved on ver. l and m. Although no differences were found between treatment conditions, attention, and ps improved only in the risperidone group
Purdon et al. (24)	Quetiapine and haloperidol	*N* = 22	6-month, randomized, double-blind clinical trial	Ver. l and m; vis. l and m; ps; r and ps	Ver. l and m, vis. l and m, and r and ps did not improve in both treatment conditions. Ps was found to improve in both treatment conditions but this was not significant after corrections for multiple testing
Velligan et al. (25)	Quetiapine and haloperidol	*N* = 58	24-week, randomized, double-blind clinical trial	Ver. l and m; ps	After Bonferroni correction, no significant improvement on ver. l and m and ps in all treatment conditions
Kivircik Akdede et al. (26)	Quetiapine	*N* = 14	8-week clinical trial	Ver. l and m; wm; ps	Improvement was found in wm and ps. Ver. l and m did not improve
Kim et al. (27)	Risperidone depot	*N* = 36	26-week, open-label trial	Ver. l and m; attention; ps; r and ps	Significant improvement in ver. l and m, attention, and r and ps. Ps did not improve
Suzuki and Gen (28)	Risperidone and haloperidol depot	*N* = 20	24-week, open-label trial	Ver. l and m; r and ps	Compared to the haloperidol group, ver. l and m and r and ps improved in the risperidone depot group
Surguladze et al. (30)	Risperidone depot and typical antipsychotics depot (haloperidol, fluphenazine, flupenthixol, zuclopenthixol)	*N* = 32 patients and 8 healthy controls	4-month clinical trial	Ver. l and m; vis. l and m	Although no differences were found between treatment conditions, both groups improved on ver. l and m and vis. l and m
Kim et al. (31)	Paliperidone and risperidone	*N* = 58	12-week, randomized open-label clinical trial	Ver. l and m; wm; attention; ps	Compared to the risperidone group, ver. l and m improved more in the paliperidone group. No differences between treatment conditions were found in wm, attention, and ps
Purdon et al. (32)	Clozapine	*N* = 18	6-week clinical trial	Ver. l and m; vis. l and m; wm; ps; r and ps	Significant improvement in ver. l and m, vis. l and m, and ps. No improvement in wm and r and ps
Ertugrul et al. (33)	Clozapine	*N* = 20 patients and 20 healthy controls	8-week clinical trial	Ver. l and m; vis. l and m; wm; r and ps	No significant improvement in ver. l and m, vis. l and m, and r and ps. Performance on one of the wm tasks improved
Sumiyoshi et al. (34)	Clozapine	*N* = 27	6-week clinical trial	Ver. l and m	No significant improvement in ver. l and m
Geffen et al. (35)	BL-1020 and risperidone	*N* = 363	6-week, randomized, double-blind, placebo-controlled clinical trial	Ver. l and m; wm; ps; r and ps	Compared to placebo, improvement in ver. l and m and wm was found with a high dose of BL-1020. Ps and r and ps did not improve
Riedel et al. (39)	Aripiprazole	*N* = 56	8-week, open-label clinical trial	Ver. l and m; vis. l and m; wm; ps	Improvement in ver. l and m and ps. Vis. l and m and wm did not improve
Bervoets et al. (40)	Aripiprazole	*N* = 238	12-week, open-label clinical trial	Ver. l and m	Ver. l and m improved significantly
Suzuki et al. (41)	Aripiprazole, perospirone, and olanzapine	*N* = 31	8-week, clinical trial	Ver. l and m; r and ps	None of the treatment conditions showed improved on ver. l and m and r and ps. No differences were found between groups
Yasui-Furukori et al. (42)	Aripiprazole (added to risperidone or olanzapine)	*N* = 36	12-week, randomized, double-blind placebo-controlled trial	Ver. l and m; wm; ps; r and ps	None of the cognitive domains measured improved significantly after adjunctive aripiprazole treatment
Rollnik et al. (69)	Atypical (olanzapine, risperidone, clozapine) and typical antipsychotics (haloperidol, chlorprothixene, perazine, flupenthixol)	*N* = 20 patients and 14 healthy controls	12-week, clinical trial	Vis. l and m; wm; ps	No significant differences were found between typical and atypical antipsychotics for all cognitive domains
McGurk et al. (70)	Risperidone and haloperidol	*N* = 27	4-week, randomized double-blind clinical trial	Vis. l and m; wm	After corrections for anticholinergic co-medication, no differences were found between risperidone and haloperidol on vis. l and m and wm
Mori et al. (75)	Risperidone, olanzapine, perospirone; quetiapine	*N* = 77	8-week, randomized clinical trial	wm	Wm improved in the risperidone group and declined in the quetiapine group. Wm performance did not change in the olanzapine and perospirone group
Papageorgiou et al. (76)	Olanzapine and clozapine	*N* = 16	6-week, randomized, double-blind clinical trial	wm	Wm improved in both groups. No significant differences between treatment conditions
Nielsen et al. (77)	Sertindole (added to clozapine)	*N* = 41	12-week, randomized, double-blind, placebo-controlled trial	Wm; r and ps	Adjunctive sertindole did not improve wm and r and ps
Molina et al. (85)	Olanzapine	*N* = 17	24-week clinical? Trial	Attention	No improvement in attention
Liu et al. (86)	Risperidone and haloperidol	*N* = 38	12-week, randomized, double-blind clinical trial	Attention	No improvement in attention
Mizrahi et al. (97)	Clozapine, risperidone, olanzapine, and loxapine	*N* = 71	6-week, clinical trial	sc	Improvement in sc
Sumiyoshi et al. (100)	Perospirone	*N* = 5	6-month, clinical trial	sc	Improvement in sc
Behere et al. (101)	Risperidone	*N* = 25 patients and 30 healthy controls	Mean duration 38 days, clinical trial	sc	Improvement in sc

*Ver. l and m, verbal learning and memory; vis. l and m, visual learning and memory; wm, working memory; ps, processing speed; r and ps, reasoning and problem solving; sc, social cognition*.

**Table 3:** **Summary of included studies using non-antipsychotic interventions**.

Study	Agent	Mechanism of action	Sample size (in analyses)	Design	MATRICS domains measured	Results
George et al. (48)	Dihydrexidine	D_1_ agonist	*N* = 20	Single-challenge, randomized placebo-controlled cross-over trial	Ver. l and m; wm; attention; ps	No improvement in any of the measured domains
Pietrzak et al. (72)	d-Amphetamine	Indirect D_1_ agonist	*N* = 32	Single-challenge, double-blind, placebo-controlled cross-over trial	Vis. l and m; ps; r and ps	Improvement in ps and r and ps. No improvement in vis. l and m
Sumiyoshi et al. (38)	Tandospirone	5-HT_1a_ agonist	*N* = 31	4-week, clinical trial	Ver. l and m; vis. l and m	Ver. l and m improved after adjunctive tandospirone treatment. Vis. l and m did not improve
Akhondzadeh et al. (43)	Ondansetron	5-HT_3a_ antagonist	*N* = 30	12-week, randomized double-blind placebo-controlled trial	Ver. l and m; vis. l and m; wm; r and ps	Vis. l and m improved significantly after adjunctive ondansetron treatment. None of the other domains improved
Levkovitz et al. (44)	Ondansetron	5-HT_3a_ antagonist	*N* = 21	2-week, double-blind placebo-controlled cross-over trial	Ver. l and m; vis. l and m; wm; ps	Significant improvement in vis. l and m in the ondansetron group. None of the others domains improved significantly
Zhang et al. (45)	Tropisetron	5-HT_3a_ antagonist	*N* = 38	10-day, randomized double-blind, placebo-controlled trial	Ver. l and m; vis. l and m; wm	Ver. l and m improved significantly after adjunctive tropisetron treatment. Other domains did not improve significantly
Niitsu et al. (71)	Fluvoxamine	σ-1 Agonist	*N* = 47	12-week, randomized double-blind placebo-controlled trial	Vis. l and m; wm; r and ps	None of the cognitive domains measured improved significantly after adjunctive fluvoxamine treatment
Friedman et al. (47)	Citalopram	Serotonin reuptake inhibition	*N* = 18	24-week, randomized double-blind placebo-controlled cross-over trial	Ver. l and m; wm; attention; ps; r and ps	None of the cognitive domains measured improved significantly after adjunctive citalopram treatment
Velligan et al. (51)	AZD3480	Nicotinic α_4_β_2_ agonist	*N* = 440	12-week, randomized double-blind placebo-controlled trial	Ver. l and m; vis. l and m; wm; attention; ps; r and ps	None of the cognitive domains measured improved
Freedman et al. (52)	DMXB-A	Nicotinic α_7_ agonist	*N* = 31	4-week, double-blind placebo-controlled cross-over trial	Ver. l and m; vis. l and m; wm; attention; ps; r and ps	No significant improvement in any of the cognitive domains over three treatment arms. After 4 weeks (one treatment arm) attention improved in both DMXB-A conditions compared to placebo
Tugal et al. (50)	Donepezil	Acetylcholinesterase inhibition	*N* = 12	12-week randomized double-blind placebo-controlled cross-over trial	Ver. l and m; vis. l and m; wm; ps; r and ps	None of the cognitive domains measured improved after adjunctive donepezil treatment
Akhondzadeh et al. (53)	Donepezil	Acetylcholinesterase inhibition	*N* = 30	12-week randomized double-blind placebo-controlled trial	Ver. l and m; vis. l and m; wm; r and ps	None of the cognitive domains measured improved after adjunctive donepezil treatment
Friedman et al. (58)	Donepezil	Acetylcholinesterase inhibition	*N* = 34	12-week randomized double-blind placebo-controlled trial	Ver. l and m; wm; attention; ps; r and ps	None of the cognitive domains measured improved after adjunctive donepezil treatment
Freudenreich et al. (57)	Donepezil	Acetylcholinesterase inhibition	*N* = 30	8-week double-blind placebo-controlled trial	Ver. l and m; wm; ps	None of the cognitive domains measured improved after adjunctive donepezil treatment
Fagerlund et al. (55)	Donepezil	Acetylcholinesterase inhibition	*N* = 11 patients and 11 healthy controls	12-week randomized double-blind placebo-controlled trial	Ver. l and m; vis. l and m; wm; ps; r and ps	None of the cognitive domains measured improved after adjunctive donepezil treatment
Lee et al. (54)	Galantamine	Acetylcholinesterase inhibition	*N* = 24	12-week, randomized double-blind placebo-controlled trial	Ver. l and m; vis. l and m; ps	Visual recognition improved in the galantamine group. None of the other measures improved significantly
Sharma et al. (56)	Rivastigmine	Acetylcholinesterase inhibition	*N* = 21	24-week, randomized double-blind placebo-controlled trial	Ver. l and m; wm; attention; ps; r and ps	No significant improvement in any of the measured cognitive domains after adjunctive rivastigmine treatment
Lenzi et al. (88)	Rivastigmine	Acetylcholinesterase inhibition	*N* = 10	52-week clinical trial	Ver. l and m; wm; attention; ps; r and ps	No significant improvement in any of the cognitive domains after adjunctive rivastigmine treatment
Duncan et al. (81)	d-Cycloserine	NMDA partial agonist	*N* = 22	4-week, randomized double-blind placebo-controlled trial	Wm; attention	Both domains did not improve significantly after adjunctive d-cycloserine treatment
Muscatello et al. (92)	Topiramate	AMPA/kainite antagonist	*N* = 43	24-week randomized double-blind placebo-controlled trial	Attention; r and ps	Both domains did not improve significantly after adjunctive topiramate treatment
Zoccali et al. (93)	Lamotrigine	Glutamate release regulation	*N* = 51	24-week randomized double-blind placebo-controlled trial	Attention; r and ps	After Bonferroni correction, no significant improvement was found in both domains
Levkovitz et al. (73)	Minocycline	Blockade nitric oxide induced neurotoxicity	*N* = 21	22-week randomized double-blind placebo-controlled trial	Vis. l and m; wm; r and ps	Vis. l and m, wm and r and ps improved after adjunctive minocycline treatment.
Buchanan et al. (59)	MK-0777	GABA α_2_/α_3_ agonist	*N* = 53	4-week randomized, double-blind placebo-controlled trial	Ver. l and m; vis. l and m; wm; attention; ps; r and ps; sc	After correction for multiple testing, none of the domains improved significantly after adjunctive MK-077 treatment
Menzies et al. (80)	Lorazepam and attention flumazenil	GABA_A_ agonist and GABA_A_ antagonist	*N* = 11 patients and 11 healthy controls	Single-challenge randomized double-blind placebo-controlled cross-over trial	wm	Wm performance declined significantly after lorazepam administration whereas no significant differences were found in the flumazenil group
Friedman et al. (60)	Atomoxetine	Norepinephrine reuptake inhibition	*N* = 20	8-week randomized, double-blind placebo-controlled trial	Ver. l and m; wm; ps; r and ps	No significant improvement in any of the cognitive domains after adjunctive atomoxetine treatment.
Turner et al. (63)	Modafinil	Unknown	*N* = 20	Single-challenge, randomized, double-blind placebo-controlled cross-over trial	Ver. l and m; vis. l and m; wm; attention; ps; r and ps	Wm improved in the modafinil condition compared to placebo. Response latency on a r and ps task was significantly lower in the modafinil condition. No differences were found in number of attempts on the same task
Spence et al. (82)	Modafinil	Unknown	*N* = 17	Single-challenge, randomized, double-blind placebo-controlled cross-over trial	wm	No significant difference between the modafinil and placebo condition
Kane et al. (61)	Armodafinil	unknown	*N* = 49	4-week, randomized double-blind placebo-controlled trial	Ver. l and m; vis. l and m; wm; attention; ps; r and ps; sc	No significant improvement in any of the cognitive domains after adjunctive armodafinil treatment
Gallagher et al. (66)	Mifepristone	Glucocorticoid antagonist	*N* = 19	2-week, randomized double-blind placebo-controlled cross-over trial	Ver. l and m; vis. l and m; wm; attention	No significant improvement in any of the cognitive domains after adjunctive mifepristone treatment.
Goff et al. (67)	Sildenafil	PDE5 inhibition	*N* = 15	Single-challenge randomized placebo-controlled cross-over trial	Ver. l and m; wm; attention	No significant improvement in any of the cognitive domains after adjunctive sildenafil treatment

*Ver. l and m, verbal learning and memory; vis. l and m, visual learning and memory; wm, working memory; ps, processing speed; r and ps, reasoning and problem solving; sc, social cognition*.

## Verbal Learning and Memory

It has been proposed that impairments in verbal learning and memory are one of the most consistent cognitive deficits seen in schizophrenia (15) and is one of the most examined cognitive domains in these patients. A majority of the 44 included studies investigated the effects of typical and atypical antipsychotic medication on verbal learning and memory.

In the past, it was assumed that atypical antipsychotics are superior to typical antipsychotics in enhancing cognition in schizophrenia due to their affinity for both dopamine D_2_ receptors and serotonin 5-HT_2A_ receptors (16, 17). However large studies such as the Clinical Antipsychotic Trials of Intervention Effectiveness (CATIE) (18) and the European First Episode Schizophrenia Trial (EUFEST) (19) did not find differences between typical and atypical antipsychotics in cognitive enhancing effects. This theory was tested by Wagner et al. (16) who compared olanzapine, a dopamine D_2_, and serotonin 5-HT_2A_ antagonist, to amisulpride, a dopamine D_2_/D_3_ antagonist in 52 patients. No significant differences were found after 8 weeks between the two groups in terms verbal memory; both groups improved on verbal learning and memory tasks. Olanzapine was compared to risperidone and haloperidol by Purdon et al. (20). Although not significant after corrections for multiple comparisons, olanzapine was found to be superior to risperidone and haloperidol in enhancing verbal learning and memory.

The atypical antipsychotic ziprasidone was found to improve verbal learning and memory after 12 weeks of monotherapy (21). However, the study did not include a control group and practice effects were not adequately controlled for. Therefore the results have to be interpreted with caution. A study comparing the effects of risperidone and haloperidol on verbal memory found that performance on a verbal memory task remained essentially unchanged in both groups (22). Since the authors did not include a non-medicated schizophrenia group but included a healthy group as control this mainly provides evidence that there is no difference between risperidone and haloperidol. Nevertheless it suggests that both medications did not cause a clinically relevant enhancement of verbal memory. However, Harvey et al. (23) compared the effects of risperidone and quetiapine and found improvement in verbal memory in both treatment groups. The dropout rate of 57% in this trial was high which may have caused a selection bias. These results are not consistent with other findings. A small study with substantial methodological shortcomings by Purdon et al. (24) found no effect of haloperidol and quetiapine. Velligan et al. (25) also compared quetiapine to haloperidol and found that patients in the quetiapine group improved to a greater extent than the patients in the haloperidol group. However, these findings were not significant after correction for multiple comparisons. Moreover, Kivircik Akdede et al. (26) did not find improvement on a verbal learning and memory task after 8 weeks of quetiapine treatment.

Kim et al. (27) found that switching from oral atypical antipsychotics to depot risperidone led to significant improvement in verbal learning and memory. However, they also did not include a control group and did not correct for possible practice effects. Furthermore a high percentage of the patients dropped out in an early stage of the study which could have caused a bias in the sample. Despite these limitations, their results are supported by Suzuki and Gen (28) who also found an improvement in verbal learning and memory in patients treated with depot risperidone compared to patients who received haloperidol treatment (depot). However, the differences between these two treatments might be caused by the high rate of anticholinergic co-medication in the haloperidol group which is well known for its adverse effects on cognition (29). Additionally, improvement in verbal learning and memory was found by Surguladze et al. (30) with both depot risperidone and typical antipsychotics (depot). Performance did not significantly differ between the groups. However, they did not control for practice effects. Therefore improvement cannot reliably be attributed to medication effects. In addition, Kim et al. (31) found that compared to patients using risperidone, verbal memory improved more in patients using paliperidone extend release (ER), an active metabolite of risperidone with an extended delivery system that decreases fluctuations in serum drug concentrations.

Hence, the theory that atypical antipsychotic medications are superior to typical antipsychotics in enhancing verbal learning and memory is not supported by these findings. Nonetheless, some of the atypical antipsychotics, especially risperidone depot, have shown beneficial effects on this aspect of cognition.

Results regarding the effects of clozapine on verbal learning and memory are inconclusive. Three studies which investigated the effects of clozapine on verbal learning and memory were found. Purdon et al. (32) reported significant improvement in verbal learning and memory after treatment with clozapine. Their results were not replicated by Ertugrul et al. (33) and Sumiyoshi et al. (34) as both of these studies did not find improvement in verbal memory in clozapine-treated patients. This discrepancy in findings could be due to methodological differences such as different test batteries and trial duration. Moreover, the study by Purdon et al. (32) did not correct for possible practice effects and since they did not include a control group, improvement in verbal memory cannot be reliably attributed to the effects of clozapine. Thus, although results are inconclusive, at present no convincing evidence is available for the effectiveness of clozapine in enhancing verbal learning and memory in schizophrenia.

Geffen et al. (35) compared the effects of 10 and 20–30 mg of BL-1020, a new antipsychotic drug, to risperidone and placebo. BL-1020 is an antagonist for D_2_ en 5-HT_2a_ receptors and agonist for GABA_A_ receptors (35). Post-mortem studies have found altered GABAergic transmission in schizophrenia, predominantly in the PFC (36, 37). Treatment with 20–30 mg BL-1020 was found to improve performance on a verbal learning memory task compared to placebo, whereas 10 mg of BL-1020 and risperidone did not have enhancing effects compared to placebo. A substantial number of patients, especially in the risperidone group, received anticholinergics as well, which could have influenced the results.

Although the findings described above do not support the theory that blocking the 5-HT_2A_ receptors improves verbal learning and memory, a role for serotonin in this domain of cognition cannot be ruled out. Sumiyoshi et al. (38) found a significant improvement on verbal memory after adjunctive tandospirone (a 5-HT_1a_ agonist) treatment compared to patients who did not receive adjunctive tandospirone. Riedel et al. (39) and Bervoets et al. (40) both found a significant improvement in verbal memory in patients treated with aripiprazole, an atypical antipsychotic drug with partial D_2_ agonistic and antagonistic and 5-HT_2a_ antagonistic properties in addition to partial agonistic activity at the 5-HT_1a_ receptors. However, this is contradicted by Suzuki et al. (41) who compared the effects of aripiprazole, olanzapine, and perospirone on verbal memory. None of the three conditions showed significant changes in verbal memory scores and no differences were found between the three treatment conditions. Moreover, aripiprazole added to atypical antipsychotics did not improve verbal learning and memory in a trial by Yasui-Furukori et al. (42). This discrepancy in findings could be due to practice effects in the studies of Riedel et al. (39) and Bervoets et al. (40), since both studies did not include a control group and did not correct for possible practice effects. Furthermore, different tasks were used which makes it difficult to directly compare the results.

The role of the serotonin 5-HT_3a_ receptor in verbal learning and memory has been examined as well. Akhondzadeh et al. (43) and Levkovitz et al. (44) investigated the effects of ondansetron, a serotonin 5-HT_3a_ receptor antagonist, added to respectively risperidone and clozapine treatment, and found no enhancing effects on verbal learning and memory. However, the sample size of both studies was small. In contrast to these results, Zhang et al. (45) did find improvement in verbal memory after add-on tropisetron (a 5-HT_3a_ receptor antagonist and nicotinic α_7_ agonist) to risperidone treatment compared to placebo. This discrepancy in findings could be explained by the fact that the two studies used different pharmacological interventions. Both Akhondzadeh et al. (43) and Levkovitz et al. (44) administered ondansetron whereas Zhang et al. (45) used tropisetron. Since ondansetron has been found to have low affinity for the nicotinic α_7_ receptors (46), the positive effects of tropisetron on verbal learning and memory could be due to its nicotinic α_7_ agonistic properties rather than its antagonistic effects on the 5-HT_3a_ receptors.

Effects of a selective serotonin reuptake inhibitor (SSRI) were examined by Friedman et al. (47) who added citalopram to treatment with atypical antipsychotics but found no improvement in verbal learning and memory.

Thus, although results are inconclusive there is preliminary evidence of positive effects of 5-HT_1a_ agonists, but not 5-HT_3a_ antagonists on verbal learning and memory in schizophrenia.

One study examined the effects of a dopamine D_1_ *agonist* on verbal learning and memory in schizophrenia. George et al. (48) administered dihydrexidine as a pharmacological challenge and found no effects of dihydrexidine on verbal learning and memory.

The role of the neurotransmitter acetylcholine in cognition has been widely investigated and established (36). Post-mortem, a correlation was found between cognitive impairment and decreased levels of brain choline acetyltransferase in schizophrenia (49). Post-mortem studies have shown changes in both muscarinic and nicotinic acetylcholine receptors in patients with schizophrenia (50). Velligan et al. (51) conducted a trial to assess the effects adjunctive therapy with the selective nicotinic α_4_β_2_ receptor agonist AZD3480 on verbal learning and memory in patients but found no improvement. An important limitation of this study is that only patients who smoked were included. Therefore, the lack of effect could be due to desensitization of the nicotinic receptors caused by chronic tobacco use. However, Freedman et al. (52) also did not find an improvement in verbal learning and memory after administration of the partial nicotinic α_7_ agonist DMXB-A, added to antipsychotic treatment in patients, who abstained from nicotine at least 1 month prior to participation. Studies using acetylcholinesterase (enzyme that breaks down acetylcholine, thereby increasing acetylcholine levels) inhibitors (AChE-Is) as adjunctive therapy to antipsychotic medication also found no improvement on verbal learning and memory (53–58). These results suggest that acetylcholinesterase inhibitors do not effectively enhance verbal memory in patients with schizophrenia. However, all studies used a small sample size and one study included patients who were taking anticholinergic medication as well. This could have influenced the outcome of the study. Another important limitation is that none of these studies controlled for the effects of smoking. Hence, although there is theoretical evidence for a role of acetylcholine in cognition, various (possibly underpowered) intervention studies have not yielded positive results. Since Zhang et al. (45) found improvement in verbal learning and memory after adjunctive tropisetron treatment, it remains possible that enhancement of verbal learning and memory can be achieved with nicotinic α_7_ agonists while simultaneously blocking the 5-HT_3a_ receptors.

The role of the neurotransmitters GABA and norepinephrine in verbal learning in memory has also been investigated. Buchanan et al. (59) found no improvement in verbal learning and memory with adjunctive MK-0777, a partial GABA α_2_/α_3_ agonist, therapy to antipsychotics. However, the authors argue that a lack of effect may be due to the fact that MK-0777 is a weak GABA α_2_/α_3_ agonist. A pilot study by Friedman et al. (60) found no effects of the norepinephrine reuptake inhibitor atomoxetine, on verbal memory when added to treatment with atypical antipsychotics.

Hence, although positive effects were found with the antipsychotic BL-1020 with GABA_A_ agonistic properties, the available studies were not able to detect positive effects of both a partial GABA α_2_/α_3_ agonist and a norepinephrine reuptake inhibitor on verbal learning and memory. Nonetheless, these results suggest that GABA_A_ receptors may be a potential target for future studies in verbal learning and memory.

Effects of the psychostimulant armodafinil were investigated by Kane et al. (61). Armodafinil is a longer-lasting isomer of modafinil, which is an alertness-promoting medication with mechanisms of action different from those of amphetamine (62) and has been found to improve cognition in healthy subjects and adults with ADHD (63). However, the exact mechanisms of action are complex and not entirely understood (64). Armodafinil, added to atypical antipsychotics, did not enhance verbal learning and memory.

The effects of several other pharmacological interventions on verbal learning and memory in schizophrenia have been investigated. Six months of daily creatine administration added to antipsychotic treatment did not have beneficial effects on verbal learning and memory (65). Effects of adjunctive mifepristone, a glucocorticoid receptor (GR) antagonist, on verbal learning and memory was examined by Gallagher et al. (66). They found no improvement in this domain of cognition. Goff et al. (67) examined the effects of sildenafil, a phosphodiesterase 5 (PDE5) inhibitor used to treat erectile dysfunction, on verbal learning and memory. PDE5 inhibitors increase cyclic guanine monophosphate (cGMP) which is thought to modulate long-term potentiation. They found single dose of 50 and 100 mg not to improve verbal learning and memory. On the other hand, Feifel et al. (15) found that patients who received intranasal oxytocin for 3 weeks performed better on a verbal learning and memory task. Additionally, Stone et al. (68) found that additional glucose administration in patients who were stable on clozapine, enhanced verbal learning and memory. All studies used a small sample and the dropout rate in the study by Feifel et al. (15) was 25%. Moreover, the majority of the patients in the study of Stone et al. (68) received additional medication besides clozapine. Therefore the effect cannot be attributed to a unique interaction of clozapine and glucose.

To summarize, the proposed superior effect on verbal learning and memory of atypical to typical antipsychotics, is not supported by the available data and any effect does not result from blocking the 5-HT_2a_ receptors. Despite the fact that multiple studies describe a role of acetylcholine in cognition, nicotinic receptor agonist and acetylcholinesterase inhibitors do not seem to effectively improve verbal learning and memory in schizophrenia. However, some positive results have been found with risperidone depot, possibly because of medication compliance. In addition, serotonin 5-HT_1a_ receptors and GABA_A_ receptors may be molecular targets for enhancing this aspect of cognition. Additionally, there is preliminary evidence that adjunctive oxytocin and glucose treatment may be beneficial in enhancing verbal learning and memory. However, results have to be interpreted with care as the available studies are mostly pilot studies which, although important in early stages of research, inherently suffer from methodological shortcomings.

## Visual Learning and Memory

Compared to verbal learning and memory, considerably less research has been conducted regarding visual learning and memory in schizophrenia. Results of studies that examined the effects of antipsychotic medication on visual learning and memory are, similar to verbal learning and memory, indecisive. Tyson et al. (17) compared the effects of atypical antipsychotic medication with high and low affinity for the 5-HT_2a_ receptors on visual learning and memory. Patients treated with risperidone, olanzapine, and clozapine were assigned to the high-affinity group and patients using quetiapine and amisulpride were assigned to the low affinity group. Performance on visual memory tasks improved in patients in the low affinity group whereas performance in the high-affinity group declined. A limitation is that the authors did not correct for multiple testing. However, the effects were already present after 9 months of treatment and show a consistent pattern. Since performance declined in the high-affinity group, it is not likely that the improvement in the low affinity group is driven by practice effects. However, Rollnik et al. (69) found atypical antipsychotics (olanzapine, risperidone, or clozapine) to be superior to typical antipsychotics (haloperidol, chlorprothixene, perazine, or flupenthixol) in improving visual learning and after 3 weeks of treatment, although at the final assessment, this difference was no longer significant. Purdon et al. (20) compared the effects of olanzapine, risperidone, and haloperidol. After correction for multiple comparisons, no significant differences were found between the three treatment conditions; visual learning and memory did not improve in all three groups. As mentioned earlier, lack of effect could be caused by concomitant anticholinergic treatment and the small sample size.

Two studies compared the effects of haloperidol to respectively quetiapine (24) and risperidone (70). In both studies haloperidol and both quetiapine and risperidone were found not to enhance visual learning and memory. However, a substantial number of patients in both studies were treated with anticholinergics in addition to antipsychotics, which could have influenced the results. Surguladze et al. (30) found no differences in visual learning and memory between depot risperidone and a typical antipsychotic depot treatment, although performance in both groups improved. As discussed earlier, it cannot be ruled out that this improvement is caused by practice effects rather than the effects of medication.

Although results are inconclusive, clozapine does not seem to effectively enhance visual learning and memory. A study by Ertugrul et al. (33) did not find improvement with clozapine whereas Purdon et al. (32) found significant improvement on performance on one of three assessed visual memory subtests. However, this study has important limitations, which were described earlier.

Treatment with aripiprazole did not significantly improve visual learning and memory in the study by Riedel et al. (39). Tandospirone, which like aripiprazole has serotonin 5-HT_1a_ agonistic properties, also did not significantly enhance visual learning and memory in the study of Sumiyoshi et al. (38). These findings suggest that stimulating the serotonin 5-HT_1a_ receptors does not enhance visual learning and memory in schizophrenia. However, the serotonin 5-HT_3a_ receptors may play a role in visual memory in schizophrenia. Both Akhondzadeh et al. (43) and Levkovitz et al. (44) found visual learning and memory to improve with adjunctive ondansetron treatment. Although both studies did not correct for multiple testing and possible practice effects, visual memory was the only aspect of the cognitive domains measured that improved in both studies. Adjunctive tropisetron treatment on the other hand did not enhance visual memory (45). As mentioned earlier, tropisetron is both a 5-HT_3a_ antagonist and partial nicotinic α_7_ agonist whereas ondansetron has low affinity for the nicotinic α_7_ receptors (46). Hence, the 5-HT_3a_ receptors may play a role in visual learning and memory. However, enhancement of this aspect of cognition may depend on blockade of nicotinic α_7_ receptors. The 5-HT_1a_ receptor does not seem to be a promising target for enhancing visual learning and memory.

A preliminary study by Niitsu et al. (71) investigated the effect of the SSRI fluvoxamine, a sigma-1 receptor agonist, on cognition and found it not to enhance visual learning and memory. These preliminary results indicate sigma-1 receptor agonism does not enhance visual learning and memory in schizophrenia patients.

Studies investigating the role acetylcholine in visual learning and memory in schizophrenia have not yielded positive results. Selective nicotinic α_4_β_2_ receptor agonist AZD3480 and partial nicotinic α_7_ agonist DMXB-A both did not enhance visual learning and memory when added to antipsychotic treatment (51, 52). As mentioned earlier, the lack of effect in the study of Velligan et al. (51) could be due to desensitization of the nicotinic receptors caused by chronic tobacco use since only smokers were included. Studies conducted with acetylcholinesterase inhibitors do not report positive results either. Although Lee et al. (54) found improvement on the recognition subtest of Rey Complex Figure Test (RCTF) with galantamine, the other aspects of the task did not improve. Studies using donepezil also did not find improvement in visual learning and memory (50, 53, 55). Hence, both nicotinic receptor agonists and acetylcholinesterase inhibitors do not seem to effectively enhance visual learning and memory in schizophrenia.

Based on preliminary results of Buchanan et al. (59), GABA does not seem to enhance visual learning and memory in schizophrenia as they found no improvement with partial GABA_A_ α_2_/α_3_ agonist MK-0777. However, this is the only study investigating the role of GABA in visual learning and memory and MK-0777 is a weak GABA α_2_/α_3_ agonist.

The possible enhancing effect of psychostimulant drugs on visual learning and memory has been investigated in schizophrenia as well. A single dose of d-amphetamine, an indirect dopamine D_1_ agonist, did not improve visual learning and memory (72). A single administration of modafinil did not significantly enhance visual memory either (63). In addition, adjunctive armodafinil treatment did not improve visual learning and memory (61). Hence, based on these findings d-amphetamine and modafinil do not seem to be promising intervention strategies for enhancing visual learning and memory.

Results of other pharmacological interventions on visual learning and memory are mixed. Gallagher et al. (66) found no improvement after adjunctive mifepristone (a GR antagonist) treatment. The effect of adjunctive creatine treatment was investigated by Kaptsan et al. (65) but they found it not to enhance visual learning and memory. Levkovitz et al. (73) investigated the effects of minocycline, a second generation tetracycline with anti-inflammatory and antimicrobial effects. Minocycline modulates the glutamate pathway by blocking nitric oxide induced neurotoxicity. Hyperactivity of glutamatergic neurotransmission [possibly caused by hypofunction of *N*-methyl-d-aspartate (NMDA) receptors] has been found in schizophrenia (36). Stimulated by glutamate, the NMDA receptors activate production of nitric oxide. The authors found improvement on a spatial recognition memory task after minocycline administration, added to antipsychotic treatment. Task performance showed a decrease after 10 weeks, but compared to baseline, performance improved at the final assessment. The drop-out rate in this study was high (due to adverse events and non-adherence) and although it did not differ between the two treatment groups, this could have biased the results.

To summarize, results regarding visual learning and memory are inconclusive. At present, there is some evidence for a role of the serotonin 5-HT_3a_ receptors in this cognitive domain. 5-HT_1a_ and sigma-1 receptor agonist on the other hand, did not yield promising results. Additionally, minocycline was found to have positive effects on visual learning memory when added to antipsychotic treatment. Both typical and atypical antipsychotics do not seem to enhance visual learning and memory. Studies investigating nicotinic receptor agonist, acetylcholinesterase inhibitors, GABA α_2_/α_3_ agonist, psychostimulants, and GR antagonist did not yield positive results but are in need of replication as they were possibly underpowered. Overall, results need be interpreted with care as the described studies are often small pilot studies with inherent limitations.

## Working Memory

Working memory refers to a system with limited capacity for temporary storage and manipulation of information, necessary for cognitive tasks (74), and has been the subject of many studies investigating cognition in schizophrenia. Most of these studies focused on the effects of antipsychotic medication on working memory.

Although results are inconclusive at present, both typical and atypical antipsychotics show little beneficial effects on working memory. Rollnik et al. (69) compared the effectiveness of typical and atypical antipsychotics and found no differences between the two groups. Quetiapine and amisulpride, which both have low affinity for the 5-HT_2a_ receptors, improved performance on a verbal working memory task compared to risperidone, olanzapine, and clozapine (high affinity for 5-HT_2a_) treatment (17). However, performance on a visual working memory tasks did not significantly improve in both groups. No differences were found in working memory performance between amisulpride and olanzapine by Wagner et al. (16). However, working memory performance did improve in both groups. Effects of olanzapine, risperidone, and haloperidol were compared by Purdon et al. (20). After correction for multiple comparisons, no significant differences were found between the three treatment conditions and working memory did not improve in any group. Mori et al. (75) examined the effects of switching from typical antipsychotics to olanzapine, quetiapine, risperidone, or perospirone on working memory and found improvement in working memory with risperidone, while working memory performance of the patients treated with quetiapine decreased. Working memory performance did not change in the olanzapine and perospirone group. It must be noted that the mean age in the sample was high (59.9 years) and correlated negatively with working memory scores. Therefore age related cognitive decline may have confounded these results. On the contrary, Kivircik Akdede et al. (26) found improvement on working memory with quetiapine. However, this study did not include a control group and did not correct for possible practice effects. Risperidone treatment was found to be superior to haloperidol in the study by McGurk et al. (70), but after correcting for anticholinergic co-medication, this effect was no longer significant. Working memory performance did not improve in both groups. Paliperidone treatment was not superior to risperidone treatment in enhancing working memory in the study by Kim et al. (31) and working memory did not improve in both groups.

Results regarding clozapine are inconclusive. Papageorgiou et al. (76) compared the effects of clozapine and olanzapine on working memory and found that working memory improved in both groups. No differences were found between the two treatment conditions. Ertugrul et al. (33) conducted two working memory tasks (Digit Span Test and the Auditory Consonant Trigram Test) and found that performance on the Digit Span test improved with clozapine treatment, whereas performance on the Auditory Consonant Trigram Test did not. Both studies did not correct for possible practice effects or multiple testing. These results are contradicted by Purdon et al. (32) who did not find improvement in working memory with clozapine treatment. This discrepancy in findings could be due to methodological shortcomings and differences such as different test batteries and trial duration.

Riedel et al. found that aripiprazole did not significantly enhance working memory (39). Aripiprazole also did not improve working memory when added to atypical antipsychotics (42). Adjunctive sertindole, an atypical antipsychotic with high affinity for the D_2_ and 5-HT_2a_ receptors, did not improve working memory in patients treated with clozapine (77). Finally, working memory did improve after 20–30 mg of the novel antipsychotic BL-1020 (a dopamine antagonist with GABA agonistic properties) compared to placebo whereas 10 mg BL-1020 and risperidone did not (35).

Hence, although results are preliminary and inconclusive, at present no convincing evidence is available for the effectiveness of both typical and atypical antipsychotics on working memory in schizophrenia. However, the antipsychotic BL-1020 was found to improve working memory, possibly through its GABA_A_ agonistic properties.

Single administration of the dopamine D_1_ agonist dihydrexidine did not enhance working memory in a trial by George et al. (48). Given the small sample size and the fact that dihydrexidine was only administered a single time, beneficial effects of long term dihydrexidine administration on working memory cannot be ruled out.

Serotonin 5-HT_3a_ receptors seem to be less important for working memory functioning. Both Akhondzadeh et al. (43) and Levkovitz et al. (44) found no improvement in working memory with adjunctive ondansetron (5-HT_3a_ antagonist) treatment. Moreover, adjunctive tropisetron treatment also did not enhance working memory (45).

In addition, Niitsu et al. (71) found no improvement in working memory after adjunctive fluvoxamine treatment. Moreover, 12 weeks of adjunctive citalopram treatment did not enhance working memory (47). Hence, these results suggest that serotonin modulation pharmacological agents do not significantly enhance working memory in schizophrenia.

Both the selective nicotinic α_4_β_2_ receptor agonist AZD3480 and the partial nicotinic α_7_ agonist DMXB-A did not enhance working memory performance (51, 52). Acetylcholinesterase inhibitors donepezil and rivastigmine did not enhance working memory either (50, 53–55, 57, 58, 77). Jacobsen et al. (78) examined the effects of a single administration of nicotine [nicotinic acetylcholine receptor agonist (79)] on working memory in tobacco using patients and tobacco using healthy subjects. Although patients performed significantly worse on the N-back task compared to healthy controls, nicotine administration did improve performance on the two-back in patients while performance of the healthy controls declined. Thus, although the acetylcholinesterase inhibitors donepezil and rivastigmine and nicotinic receptor agonists do not show positive effects on working memory in schizophrenia, positive results were found with a single dose of nicotine administration. Therefore, a role of acetylcholine and nicotinic receptors in enhancing working memory cannot be ruled out.

Working memory did not improve in a study by Buchanan et al. (59) after 4 weeks of treatment with partial GABA α_2_/α_3_ agonist MK-0777. Menzies et al. (80) studied the effects of lorazepam, a GABA_A_ receptor agonist, and flumazenil, a GABA_A_ receptor antagonist, on working memory in patients and healthy subjects. Working memory performance declined after a single dose of lorazepam whereas performance did not change significantly after flumazenil. Thus, although results are inconclusive, there is some evidence that in GABA_A_ agonists may lower working memory. However positive effects on working memory were found with an antipsychotic with additional GABA_A_ agonistic properties.

Friedman et al. (60) found no effects of atomoxetine (norepinephrine reuptake inhibitor) on working memory in patients. A lack of effect could be due to insufficient power.

Glutamatergic pathways have been examined by Duncan et al. (81). They added 50 mg of d-cycloserine, an antituberculous drug which, at low doses has agonistic properties at the NDMA receptors, to typical antipsychotics. After 4 weeks of treatment, working memory did not improve. However, these results need to be replicated before the role of NMDA receptors in working memory can be established.

Armodafinil, a longer-lasting isomer of modafinil, did not enhance performance on working memory task when added to atypical antipsychotics for 4 weeks (61). However, Turner et al. (63) found that a single dose of modafinil improved performance on a verbal working memory task compared to placebo. Performance on a spatial working memory task did not differ between modafinil condition and placebo condition. These results were not replicated by Spence et al. (82) who did not find differences in performance on a (verbal) working memory task after a single administration of modafinil compared to placebo. This discrepancy in findings could be due to differences in modafinil dose (200 and 100 mg, respectively). Moreover, both studies used different tasks which make direct comparison of results difficult. Nonetheless, a single dose of modafinil might enhance working memory in schizophrenia patients, although recurrent treatment with a longer-lasting variant showed no effect.

Add-on intranasal oxytocin treatment did not improve working memory (15). Furthermore, adjunctive mifepristone (GR antagonist) treatment did not enhance working memory (66). Goff et al. (67) did not find improvement in working memory after both 50 and 100 mg sildenafil administration. Furthermore, daily creatine administration added to antipsychotic treatment did not have beneficial effects on working memory either (65). Add-on minocycline treatment on the other hand, improved working memory in a study by Levkovitz et al. (73); the number of errors on a working memory task decreased significantly in the minocycline group whereas the number of errors in the placebo group increased. However, this study has some limitations which are described earlier. Thus, apart from minocycline, other lines of research did not identify possible new intervention strategies for working memory enhancement in schizophrenia.

To summarize, even though some positive results were found with minocycline, nicotine, and modafinil, the presently available studies did not identify promising molecular targets for enhancement of working memory in schizophrenia. GABA agonists have shown mixed results. Most of the studies examined the effects of antipsychotic treatment on working memory and only a limited number of studies used other pharmacological interventions, which makes it difficult to draw definite conclusions. More research needs to be conducted, especially on the potential role of GABA, norepinephrine, acetylcholine, glutamate, and psychostimulants.

## Attention and Vigilance

The construct of attention refers to a core cognitive function that relates to the ability to select, filter, focus, and process different stimuli in the environment. Attention is closely related to working memory and executive functioning (83) and therefore, it is difficult to distinguish between these constructs. Because of the broad definition, impairment in virtually any task can be considered as impairment in attention (83). Therefore, in this review, we only included articles which used the Continuous Performance Test (CPT) to specifically measure attention as this test is recommended by the MATRICS (84).

Studies using antipsychotics to enhance attention report little beneficial effects. The theory that atypical antipsychotics are superior to typical antipsychotics in enhancing cognition was tested for attention as well. A trial comparing olanzapine to amisulpride did not find differences in effects on attention; attention did not significantly improve in both groups (16). Olanzapine did not enhance attention in a study by Molina et al. (85), who examined the effects of switching from typical antipsychotics or risperidone to olanzapine. Attention did not improve with olanzapine treatment.

The effects of risperidone on attention were compared to the effects of respectively haloperidol (86) and quetiapine (23). In the study of Liu et al. (86) no improvement was found in both the haloperidol and the risperidone group. Additionally, although no differences were found between the risperidone and quetiapine in the study by Harvey et al. (23), within group analyses showed significant improvement in attention in the risperidone group, whereas the quetiapine group did not improve. Kim et al. (27) reported improvement with depot risperidone. However, paliperidone did not improve attention (31). To summarize, both typical and atypical antipsychotics have shown little beneficial effects on attention in schizophrenia patients.

The possible role of dopamine and serotonin in attention has also been examined with intervention strategies other than antipsychotic treatment. George et al. (48) examined the effects of a full dopamine D_1_ agonist, dihydrexidine but found it not to enhance attention.

Studies using serotonin intervention strategies have not yielded positive results either. Golightly et al. (87) examined the role of serotonin in attention by using acute tryptophan, a precursor for serotonin, depletion. Depletion had no effect on performance on the CPT. Niitsu et al. (71) investigated the effect of adjunctive fluvoxamine treatment on attention and found no improvement in attention either. The effects of citalopram, added to treatment with atypical antipsychotics was examined by Friedman et al. (47). They found that adjunctive citalopram treatment did not enhance attention. Thus, although serotonin has been implicated in both verbal and visual memory, it does not seem to play a prominent role in attention.

Based on results of mostly preliminary studies, acetylcholine does not seem to play an important role in attention. Selective nicotinic α_4_β_2_ receptor agonist AZD3480, did not enhance attention in a study by Velligan et al. (51). Nonetheless, Freedman et al. (52) found some beneficial effects of partial nicotinic α_7_ agonist DMXB-A on attention. In their cross-over study, patients were treated with 75, 150 mg DMXB-A, and placebo. All treatment arms lasted 4 weeks, followed by a 1-week washout period. Over the course of the trial, attention improved in all treatment groups. The authors suggested that possible practice effects had obscured the potential effect of treatment, and therefore also examined the scores after the first 4 weeks of treatment. They found that attention improved with both doses of DMXB-A compared to the placebo. Two types of acetylcholinesterase inhibitors, rivastigmine, and donepezil, did not enhance attention in schizophrenia (56, 58, 88). On the other hand, a single nicotine administration improved attention in schizophrenia patients but not in healthy controls (89). Thus, despite negative results with acetylcholinesterase inhibitors, some promising results have been obtained with nicotine and a partial nicotinic α_7_ agonist. These preliminary results suggest that the nicotinic receptors are a potential target for enhancement of attention in schizophrenia.

Partial GABA α_2_/α_3_ agonist MK-0777 did not enhance attention when added to antipsychotic treatment (59). However, as mentioned earlier, MK-0777 is a weak GABA α_2_/α_3_ agonist and this is the only study investigating the potential role of GABA in attention in schizophrenia. Therefore, these results need to be replicated using specific and more potent GABA modulatory agents.

Other lines of research have not yielded positive results. The psychostimulant drug armodafinil did not enhance attention (61). Furthermore, adjunctive mifepristone (GR antagonist) therapy did not improve attention (66). Duncan et al. (81) found no improvement in attention with adjunctive d-cycloserine treatment. Also, sildenafil (PDE5 inhibitor) did not enhance attention in schizophrenia in a trial by Goff et al. (67). Stone et al. (68) compared the effects of single administration of glucose to placebo in clozapine-treated patients and found attention to be significantly worse in the glucose condition. Thus, several other lines of research did not identify potential new intervention strategies enhancement of attention in schizophrenia.

To summarize, at present only a few pharmacological intervention strategies have been effective in enhancing attention in schizophrenia. Although results are not consistent, positive results have been found with both oral and depot risperidone treatment. Furthermore, a partial nicotinic α_7_ agonist, and a single administration of nicotine did improve attention in schizophrenia, suggesting a role for acetylcholine and nicotinic receptors in attention. Overall, results need to be considered preliminary and more research needs to be conducted to replicate these findings as these studies have some important limitations.

## Processing Speed

Processing speed is considered a core cognitive function and refers to the speed at which the brain processes information. It can be measured as the number of correct responses during a task within a given amount of time (90).

As for other domains of cognition, a majority of the studies examined the effects of antipsychotic medication on processing speed. The theory that atypical antipsychotics are superior to typical antipsychotics in improving cognition in patients was tested for processing speed as well. A study comparing amisulpride with olanzapine found that processing speed did not improve in both groups (16). Moreover, performance on a processing speed task did not differ between patients treated with different typical antipsychotics and atypical antipsychotics and performance did not improve in both groups (69). Purdon et al. (20) compared the effects of olanzapine, risperidone, and haloperidol on processing speed. After correction for multiple comparisons, no significant differences were found between the three treatment conditions and processing speed did not improve in any of the groups. Thus, atypical antipsychotics do not appear to be superior to typical antipsychotics. Furthermore, no beneficial effects of the atypical antipsychotic ziprasidone were found on processing speed (21). Anticholinergics were allowed during the trial which might have influenced the results. Risperidone depot treatment did not enhance processing speed in the study by Kim et al. (27). Moreover, paliperidone ER was not found to be superior to risperidone (31). Unfortunately, within group comparisons were not reported. However, although no significant differences were found between two treatment groups, risperidone was found to improve processing speed whereas quetiapine did not in a study comparing these two antipsychotics (23). Contrary to those results, quetiapine was found to enhance processing speed in a trial by Kivircik Akdede et al. (26). As described earlier, this study did not include a control group and did not correct for possible practice effects. These results were not replicated by Purdon et al. (24). Although processing speed improved in both the quetiapine and haloperidol group, these results were not significant after correcting for multiple comparisons. Velligan et al. (25) compared the effectiveness on processing speed between quetiapine and haloperidol treatment. They found no differences between the two treatment groups. Purdon et al. (32) found that processing speed improved with clozapine treatment. However, this improvement cannot reliably be attributed to the effects of clozapine as the study did not include a control group en they did not correct for possible practice effects. Beneficial effects of aripiprazole on processing speed were found by Riedel et al. (39). Yasui-Furukori et al. (42) found no improvement in processing speed with adjunctive aripiprazole treatment to olanzapine or risperidone. Furthermore, a new antipsychotic with GABA_A_ receptor agonistic properties in addition to dopamine D_2_ and serotonin 5-HT_2a_ receptor blockade (BL-1020) did not improve processing speed (35).

To summarize, both typical and atypical antipsychotics do not seem to effectively enhance processing speed in schizophrenia patients. Although some studies reported positive effects of clozapine, quetiapine, and risperidone, these results should be interpreted with care as these studies have important limitations.

Studies examining the role of serotonin in processing speed did not yield positive results. Adjunctive ondansetron (5-HT_3a_ receptor antagonist) therapy to antipsychotic medication did not enhance processing speed in a study by Levkovitz et al. (44). Adding citalopram to atypical antipsychotic treatment also did not enhance processing speed in the study by Friedman et al. (47). In addition, tryptophan depletion did not affect processing speed in the study by Golightly et al. (87). Thus, the available studies do not provide evidence for a prominent role of serotonin in processing speed in schizophrenia. However, all studies used small samples and the study of Golightly et al. (87) allowed concomitant anticholinergics which could have influenced the results.

Studies using acetylcholine related intervention strategies did not yield positive results either. Both the selective nicotinic α_4_β_2_ receptor agonist AZD3480 and the partial nicotinic α_7_ agonist DMXB-A did not enhance processing speed (51, 52). Moreover, three types of acetylcholinesterase inhibitors, donepezil, galantamine, and rivastigmine, did not improve processing speed (50, 54–58). Thus, although the role of acetylcholine in memory and attention has been well established, it does not seem to be a potential target in enhancing processing speed in schizophrenia. However, these studies have limitations such as small sample sizes.

Effects of GABA and norepinephrine on processing speed were also examined. Processing speed did not improve after adjunctive treatment with partial α_2_/α_3_ agonist MK-0777 (59). As mentioned earlier, BL-1020 (antipsychotic with GABA_A_ agonistic properties) enhanced antipsychotic also did not enhance processing speed (35). In the pilot study of Friedman et al. (60), processing speed did not improve after adjunctive treatment with the norepinephrine reuptake inhibitor atomoxetine. Thus, norepinephrine and GABA α_2_/α_3_ receptors do not seem to play an important role in processing speed in schizophrenia as the available studies were not able to detect positive effects. However, lack of effect could be due to the small sample size of both studies.

Processing speed did increase after a single dose of d-amphetamine in the study of Pietrzak et al. (72). Adjunctive armodafinil treatment had no beneficial effects on processing speed in the study by Kane et al. (61). Hence, since d-amphetamine is an indirect dopamine D_1_ agonist, dopamine D_1_ receptors could be a possible target for enhancing processing speed in schizophrenia although the study by Pietrzak et al. (72) needs replication.

Lastly, there are some studies of agents that have no direct (known) effect on neurotransmission. Processing speed did not improve after single dose of 50 and 100 mg sildenafil (a PDE5 inhibitor commonly used for erectile dysfunction) in the cross-over trial by Goff et al. (67). Furthermore, adjunctive dehydroepiandrosterone (DHEA) therapy, a corticosteroid that serves as a precursor for both androgenic and estrogenic steroids, did not enhance processing speed (91). Thus, other lines of research did not identify promising intervention strategies for enhancement of processing speed in schizophrenia.

To summarize, at present no convincing evidence exist for the effectiveness of both typical and atypical antipsychotics on processing speed in schizophrenia. Although, positive results were found with quetiapine, risperidone, clozapine, and aripiprazole, these studies have important limitations and therefore the effects cannot be reliably attributed to the effects of the medication. One positive result was found with single dose d-amphetamine, suggesting a potential role of dopamine D_1_ receptors in processing speed. Studies investigating 5-HT_3a_ receptor antagonist, SSRI, nicotinic receptor agonist, acetylcholinesterase inhibitors, a GABA α_2_/α_3_ agonist, and a norepinephrine reuptake inhibitor did not find clinically relevant improvement in processing speed but are in need for replication as the described studies used small samples and were possibly underpowered.

## Reasoning and Problem Solving

We found 30 studies that measured reasoning and problem solving after a pharmacological intervention. Reasoning and problem solving is considered an aspect of executive functioning. As for the other cognitive domains, a relatively large number of studies investigated the effects of antipsychotics on reasoning and problem solving.

Tyson et al. (17) compared antipsychotics with low affinity for 5-HT_2a_ receptors to antipsychotics with high affinity for these receptors. Response latency on a reasoning and problem solving task decreased in the low affinity group, whereas latency increased in the high-affinity group. The authors did not correct for multiple comparisons. Since response latency increased in the high-affinity group, it is not likely that the improvement in the low affinity group is (only) driven by practice effects. Purdon et al. (20) compared the effects of olanzapine, risperidone, and haloperidol and found that olanzapine was superior in enhancing reasoning and problem solving. However, after correcting for multiple comparisons, none of the medications improved reasoning and problem solving. On the contrary, both Kim et al. (27) and Suzuki and Gen (28) found improvement in performance on a reasoning and problem solving task. However, Kim et al. (27) did not include a control group and did not correct for possible practice effects. Therefore, improvement cannot be reliably attributed to the effects of medication. Moreover, in the study of Suzuki and Gen (28) a high percentage of the patients in the haloperidol group was using concomitant anticholinergics whereas in the risperidone group anticholinergics were tapered during the first weeks of the trial. Therefore improvement in this group could be due to effects of diminishing anticholinergics.

Purdon et al. (24) investigated the effects of haloperidol and quetiapine and found that reasoning and problem solving did not improve in both groups. Olanzapine, perospirone, and aripiprazole did not improve reasoning and problem solving either (41). Moreover, adding aripiprazole to atypical antipsychotics also did not yield positive results (42). Furthermore, no improvement was found with clozapine by Ertugrul et al. (33) and Purdon et al. (32). Nielsen et al. (77) added sertindole to clozapine but found no improvement in reasoning and problem solving either. Finally, the new antipsychotic with GABA agonistic properties BL-1020 also did not improve reasoning and problem solving (35). Hence, although positive results were found with risperidone depot, both typical and atypical antipsychotics have shown little clinically relevant effects on reasoning and problem solving.

Serotonergic intervention strategies have not yielded positive results. Adjunctive ondansetron (5-HT_3_ antagonist) treatment had no beneficial effects on reasoning and problem solving (43). Fluvoxamine and citalopram both did not enhance reasoning and problem solving either (47, 71). On the contrary, Golightly et al. (87) found that patients who underwent tryptophan depletion during the first session performed significantly worse on the Wisconsin Card Sorting Test (WCST) compared to placebo. However, this effect was not present during the second session. The WCST requires subjects to sort cards by a certain parameter. The subject is not told which parameter. The sorting principle changes after 10 correct responses in a row. Thus subjects have to acquire a certain strategy to sort the cards. The authors concluded that tryptophan depletion only affected strategy acquisition and that once this is learned; tryptophan depletion did not interfere with application of this strategy. Thus, based on the results described above, 5-HT_3a_ antagonist and SSRI’s did not effectively enhance reasoning and problem solving. However, a role for serotonin in this aspect of cognition cannot be ruled out since tryptophan depletion did interfere with strategy acquisition.

Partial nicotinic α_7_ agonist DMXB-A had no enhancing effects on reasoning and problem solving in the study by Freedman et al. (52). In addition, studies investigating the effects of the acetylcholinesterase inhibitors donepezil and rivastigmine did not find improvement in performance on a reasoning and problem solving task either (50, 53, 56, 58). Hence, based on these results, nicotinic α_7_ agonists and acetylcholinesterase inhibitors do not appear to significantly enhance reasoning and problem solving in schizophrenia.

In line with the results of Geffen et al. (35) described earlier, no positive results were obtained with other GABAergic interventions strategies. Adjunctive therapy with partial GABA α_2_/α_3_ agonist MK-0777 did not enhance reasoning and problem solving abilities in the study by Buchanan et al. (59). In addition, adjunctive topiramate (an antiepileptic drug which potentiates GABAergic transmission probably through its AMPA/kainite receptor antagonistic properties) treatment did not enhance reasoning and problem solving either (92). Zoccali et al. (93) examined the effects of adjunctive lamotrigine, an anticonvulsant drug which reduces excessive glutamate release in the brain via inhibition of voltage-gated sodium and calcium channels (94). Reasoning and problem solving did not improve.

The potential role of norepinephrine in reasoning and problem solving in schizophrenia was examined in a pilot study by Friedman et al. (60) by adding atomoxetine to antipsychotic treatment. They found no improvement in this aspect of cognition.

In conclusion, the available evidence suggests that modulation of GABA and glutamate transmission and norepinephrine reuptake inhibition does not have enhancing effects on reasoning and problem solving abilities in schizophrenia.

Some positive results have been found with psychostimulant drugs. Compared to placebo, performance on a reasoning and problem solving task improved after a single dose of d-amphetamine [indirect dopamine D_1_ agonist, (72)]. Contrary to these results, Turner et al. (63) found that, compared to placebo, response latency on the Tower of London task was significantly lower after modafinil administration. The number of attempts to obtain the correct solution did not differ between the two groups. Armodafinil, the longer-lasting isomer of modafinil, did not affect reasoning and problem solving abilities (61). Thus, although armodafinil and modafinil did not enhance reasoning and problem solving, d-amphetamine did improve this aspect of cognition. This suggests that dopamine D_1_ agonists are a potential area of research for further study of reasoning and problem solving in schizophrenia.

Pharmacological intervention strategies that do not directly influence neurotransmission were also investigated. Levkovitz et al. (73) found that reasoning and problem solving abilities improved after add-on minocycline treatment whereas performance in the placebo group did not change. Both creatine and glucose had no enhancing effects on reasoning and problem solving; daily creatine administration in addition to antipsychotic treatment did not have beneficial effects on working memory in the study by Kaptsan et al. (65). Stone et al. (68) found no improvement in reasoning and problem solving after a single dose of glucose. Hence, despite of the study’s limitations, minocycline’s putative ability to improve reasoning and problem solving abilities should be studied further.

In conclusion, although positive results were found with depot risperidone treatment in studies with important limitations, antipsychotics do not seem to effectively improve reasoning and problem solving in schizophrenia. The available studies did not show enhancing effects of the neurotransmitters serotonin, acetylcholine, GABA, or norepinephrine on reasoning and problem solving. However, dopamine D_1_ agonists may have potential in this cognitive domain since one positive result was found with a single dose d-amphetamine. Finally, minocycline had enhancing effects on reasoning and problem solving abilities. However, this study has important limitations. Therefore these results are in need for replication.

## Social Cognition

In general terms, social cognition refers to the cognitive processes used to decode and encode the social world (95). Of all the MATRICS cognitive domains, social cognition has received the least attention in research. This is probably due to the fact that it is a relatively new area in schizophrenia research (96) and that the boundaries of this domain are not entirely clear (96). Final screening yielded no more than seven articles on social cognition and pharmacology using validated outcome measures.

A study by Mizrahi et al. (97) examined the effects of antipsychotic treatment on social cognition in schizophrenia. They investigated the effects of clozapine, risperidone, olanzapine, and loxapine on Theory of Mind (TOM). TOM is an aspect of social cognition which refers to the ability to understand intentions of others and to recognize that their actions are guided by beliefs about the world (97). They found that TOM improved after 2 weeks of medication use and continued to improve during the rest of the trial. Unfortunately, the authors did not differentiate between the four different antipsychotics. However, all four types of medication have high affinity for dopamine D_2_ receptors as well as the 5-HT_2a_ receptors (98, 99). These results are in line with the results of Sumiyoshi et al. (100) who found that perospirone treatment improved performance on a social cognition task. Nonetheless, both studies did not include a control group and used small samples. Hence, given the longitudinal design of this study, natural progression and practice effects cannot be excluded. Additionally, the high drop-out rate in the study of Sumiyoshi et al. (100) could have led to a bias in the sample. Behere et al. (101) found that performance on an emotion recognition task improved after risperidone treatment. However, it must be noted that they did not include a control group and that time of follow-up assessment was not equal for all patients. Opposed to these results, the study by Harvey et al. (23) discussed earlier did not find improvement in performance on an emotional recognition task in patients treated with either quetiapine or risperidone.

Hence, these results imply preliminary evidence that dopamine and serotonin are important for TOM related aspects of social cognition but not for emotion recognition.

The possible role of GABA in social cognition was investigated by Buchanan et al. (59). They found no improvement with the partial GABA α_2_/α_3_ agonist MK-0777 when added to antipsychotic treatment. The psychostimulant armodafinil also did not improve social cognition (61). Hence, GABA α_2_/α_3_ receptors are not prime candidates for enhancing social cognition in schizophrenia.

Another line of research focused on the role of oxytocin in social cognition. This neuropeptide is known for its role in positive social behavior (102). Pedersen et al. (103) found that intranasal oxytocin administration improved social cognition. This implicates a role for oxytocin in social cognition. Since previous research showed that plasma oxytocin levels are lower in schizophrenia compared to controls (104), it could be hypothesized that increasing oxytocin levels improves social cognition in schizophrenia. However, the study of Pedersen et al. (103) has some important limitations. First, the sample size was small. Moreover, they carried out multiple tests in this small sample; therefore the results have to be interpreted with caution. Studies investigating the role of oxytocin in social cognition are sparse. Therefore, to determine the exact role of oxytocin in social cognition more research needs to be conducted on this topic.

In conclusion, the available seven studies suggest preliminary evidence for a role of dopamine, serotonin, and oxytocin in social cognition in schizophrenia patients. GABA does not seem to be a promising target for enhancement of social cognition. However, these studies have important limitations. Therefore future research needs to confirm the role of these transmitters in social cognition.

## Discussion

With this review we aimed to provide an outline of the underlying neuropharmacological mechanisms of the separate MATRICS domains. Although some potential targets were identified, overall, results of previous studies attempting to identify potential pharmacological targets for cognitive enhancement have been unsatisfactory. This review has shown that dysfunction in separate cognitive domains seems to arise from different underlying neuropharmacological mechanisms. This suggests that schizophrenia patients with different cognitive impairments could benefit from different (adjunctive) pharmacological agents. The identified potential molecular targets, which include dopamine D_1_ receptors, serotonin 5-HT_1a_ and 5-HT_3a_ receptors, nicotinic α_7_ receptors, GABA_A_ receptors, and NMDA receptors, are described more extensively below.

### Dopamine D_1_

Although George et al. (48) were not able to detect positive results with a single dose of D_1_ agonist dihydrexidine, Pietrzak et al. (72) found improvement in both processing speed and reasoning and problems solving abilities (both aspects of executive functioning) after a single dose of d-amphetamine, an indirect D_1_ agonist. Decreased dopaminergic neurotransmission in the PFC has been hypothesized to be associated with cognitive dysfunction in schizophrenia (36). Since D_1_ receptors are highly abundant in the PFC (105), this receptor subtype has been particularly associated with executive function and working memory. A PET study by Okubo et al. (106) found decreased D_1_ receptors in the PFC in schizophrenia which was indeed associated with poorer executive functioning. Animal studies provide additional evidence for cognitive enhancing effects of D_1_ receptor agonists as low doses of several D_1_ agonists were found to enhance cognition in non-human primates (107, 108). Because a majority of dopamine receptors in the PFC belong to the D_1_ subtype, and not to the D_2_ subtype (109), which is related to psychotic symptom severity, D_1_ receptors may be a feasible molecular target for enhancement of executive function related aspects of cognition without exacerbation of psychotic symptoms.

### Serotonin 5-HT_1a_ and 5-HT_3a_ receptors

(Partial) 5-HT_1a_ receptor agonism was found to improve verbal learning and memory (38–40). These enhancing effects may be due to the high density of 5-HT_1a_ receptors in the hippocampus (36), which is an area in the brain well known for its role in memory. In addition, preclinical studies showed that both 5-HT_1a_ agonists and antagonist enhanced cognition in rats (110, 111). 5-HT_3a_ receptor antagonism was associated with improvement in visual learning and memory (43, 44). Contrary to other subtypes of serotonin receptors, the 5-HT_3a_ receptor is the only ligand-gated ion channel subtype (43). To date, not many studies investigated the potential role of 5-HT_3a_ receptors in cognition. However, since both Akhondzadeh et al. (43) and Levkovitz et al. (44) found improvement in visual learning and memory with the 5-HT_3a_ receptor antagonist ondansetron, these receptors might be a promising molecular target for enhancement of visual learning and memory in schizophrenia.

### Acetylcholine nicotinic α_7_ receptors

The role of acetylcholine in cognition (particularly in learning, memory, and attention) has been widely established and central dysfunction of the cholinergic system has been found to be associated with cognitive symptoms in neurological diseases as Alzheimer and Parkinson’s disease (112–114). However, with the exception of positive results found with nicotine and a partial nicotinic α_7_ agonist on attention (52, 89), cholinergic interventions strategies did not affect any of the cognitive domains in the available studies. However, all the available studies that met the inclusion criteria used acetylcholinesterase inhibitors or nicotinic agonists. Although (mostly post-mortem) both nicotinic and muscarinic receptor abnormalities have been repeatedly found in schizophrenia (115–120), nicotinic receptor antagonists do not appear to impair cognition in the same manner as antimuscarinic drugs (121). This might explain the lack of improvement in the described studies. Indeed, a small pilot study by Shekar et al. (122) found that the muscarinic receptor agonist xanomeline improves verbal, visual, and working memory in patients with schizophrenia or schizoaffective disorder. Moreover, xanomeline improved cognition in Alzheimer disease (123). Thus, although positive effects on attention were found with nicotine and a partial nicotinic α_7_ agonist, future studies should focus on muscarinic agents.

### GABA_A_ receptors

Both GABA_A_ receptor antagonists and agonists were found to affect working memory and verbal learning and memory (35, 80). Multiple studies have shown reduced GABAergic transmission, especially in the PFC, in schizophrenia (124). The PFC is strongly involved in working memory functioning (125) and animal studies have shown that appropriate GABA transmission in the dorsolateral prefrontal cortex (DPFC) is essential to adequate working memory functioning (36, 126). Lewis et al. (37) showed that altered GABA transmission in the DPFC is possibly limited to certain cell classes, such as the chandelier cells, which synchronize the activation of the pyramidal neurons via the GABA_A_ receptor subtypes. Therefore, GABA_A_ receptors may be a promising molecular target for enhancement of working memory.

### Glutamate NMDA receptors

Glutamate, the primary excitatory neurotransmitter in the mammalian brain, has been linked to learning and memory because of its principal role in modulating long-term potentiation (127), and hyperactivity of glutamatergic neurotransmission has been implicated in schizophrenia (36). It has been hypothesized that cognitive dysfunction in schizophrenia is due to hypofunction of the NMDA receptor (81). Small increases in NMDA-dependent glutamate transmission might enhance cognition, whereas excessive stimulation might have neurodegenerative consequences (36). Activation of the NMDA receptors leads to synthesis of nitric oxide, which is able to further increase the excitotoxicity by increasing glutamate release from presynaptic neurons and inhibition of glial glutamate transporters (73). Indeed, minocycline (which blocks nitric oxide induced neurotoxicity) was found to improve visual learning and memory, working memory, and reasoning and problem solving abilities (73). Although d-cycloserine (which non-competitively enhances NMDA neurotransmission) was not found to improve attention and working memory (81), pharmacological agents reducing the neurotoxic effects of extensive glutamate might still be a promising intervention strategy for cognitive enhancement.

## Can the Negative Results be Explained by Studies Limitations?

A substantial number of the studies evaluated in this review addressing pharmacological cognitive enhancement in schizophrenia report negative results. It is debatable whether this is due to ineffectiveness of the agents used, or that potential results were obscured by methodological shortcomings as cognition research in schizophrenia deals with pertinent limitations. First, in many of the studies included in this review, cognition was not the primary outcome parameter. As a result, the design of these studies was not always optimal to measure cognitive enhancement. Second, the sample sizes are often too small to detect clinically relevant effects. Third, concurrent medications may interfere with the investigated pharmacological agents. Patients with schizophrenia are generally treated with antipsychotics. Because the entire mechanism of action of these medications is not completely understood, it cannot be ruled out that antipsychotics alter the effects of the added agents (62). Moreover, patients often use concomitant medications such as benzodiazepines, antidepressants, and anticholinergics, which could influence the effects of the adjunctive pharmacological agents as well. Especially anticholinergic medication is well known for its adverse effects on cognition (29). Therefore, studies including only medication-naïve patients at early stages of the disease are necessary. Fourth, studies often do not use fixed doses. It is possible that certain agents are only effective in a certain dose. If studies do use a fixed dose, this dose is often established for treating the illness or symptoms it was originally used for. However, it is not necessarily so that the same dose is required for cognitive enhancement in schizophrenia. Fifth, research seems to focus on the direct or semi-direct enhancing effect of modulating certain receptors while cognitive decline has most often been a process of a longer period of time possibly implying that enhancement studies should equally allow for more time to yield effects. Also, different paradigms could focus on neuroprotective targets and preventing cognitive decline early in the disorder in contrast to enhancing cognition after impairment. Sixth, not all studies report information about substance use of the participants. Patients with schizophrenia often abuse substances as tobacco, alcohol and cannabis, and epidemiological studies found high co-morbidity rates of substance abuse (40–60%) (128, 129). Particularly tobacco use can interfere with nicotinic receptor agents as it causes desensitization of this receptor subtype. Although most of the studies excluded patients with a (recent) co-morbid diagnosis of alcohol or drug abuse/dependence, tobacco using patients are almost never excluded. Seventh, studies often use different neuropsychological tasks to measure the same aspects of cognition which makes it difficult to compare the results. Therefore, studies should use a standardized cognitive battery, such as the MATRICS Consensus Cognitive Battery [MCCB; (84)] or the CANTAB cognitive battery (130). The MCCB in particular, has been composed to reliably assess cognition in schizophrenia patients. At present, only three of the reported studies used this battery to measure cognition (50, 52, 59). Finally, not all studies used a control group and repeatedly conducted the same test battery without adequately correcting for potential practice effects. Therefore, improvement often cannot be reliably attributed to the effect of the pharmacological agent. Future research should take these limitations in to account in experimental setup and optimize their design for cognition measurement. Ideally, future studies should include a placebo or other control group and should ensure sufficient power by including enough participants. Furthermore, medication-naïve patients should be recruited, preferably non-smokers who do not use other substances as cannabis and alcohol. Regarding the design, future studies should choose cognition as primary outcome measure and optimize the design by using a fixed dose and make sure to choose a dose suitable for schizophrenia patients. Finally, future studies should make sure to choose sufficient trial duration and to use a standardized cognitive test battery which covers all the MATRICS domains. In this manner, studies will be better comparable and the effects of possible confounding variables will be limited. To summarize, the negative results of “pharmacological cognitive enhancement studies” could partially be explained by studies’ limitations.

## Conclusion

Although some potential targets were identified, overall, results of previous studies attempting to identify potential pharmacological targets for cognitive enhancement have been disappointing. This review has shown that dysfunction in separate cognitive domains may arise from different underlying neuropharmacological mechanisms which suggests that schizophrenia patients with different cognitive impairments could benefit from different intervention strategies. Although development of effective cognitive enhancers is a complex process, it is an exciting challenge for this area of research as improvement of cognition contributes significantly to the quality of life of these patients.

## Author Contributions

Wilhelmina A. M. Vingerhoets wrote the first draft of the manuscript. All authors contributed to and have approved the final manuscript.

## Conflict of Interest Statement

The authors declare that the research was conducted in the absence of any commercial or financial relationships that could be construed as a potential conflict of interest.
